# Supplementary material for: Are carbon emissions trading and green financial instruments synergistic? -Comprehensive quantitative research based on content analysis
Source: PLoS One. 2024 Mar 7;19(3):e0298601. doi: 10.1371/journal.pone.0298601 (PMC10919601; doi:10.1371/journal.pone.0298601)
Supplement: S1 Data — (ZIP) [file pone.0298601.s001.zip › Data/地级市Z_F工作报告.zip!/╔╜╢1⁄2/┴─│╟╩╨/└┤╫╘╣1⁄2╓┌║┼í╛┬φ┐╦╩2╛▌═°í┐_2005.docx]

各位代表：

现在，我代表市人民政府，向大会作政府工作报告，请予审议，并请市政协各位委员和其他列席会议的同志提出意见。

一、2004年工作回顾

2004年，是我市改革开放和现代化建设取得显著成就的一年。在胡锦涛总书记视察聊城的巨大鼓舞下，在省委、省政府和市委的坚强领导下，全市各级政府同广大人民群众一道，坚持以邓小平理论和“三个代表”重要思想为指导，认真贯彻党的十六大和十六届三中、四中全会精神，全面落实科学发展观和国家宏观调控政策，按照省、市委工作会议提出的发展目标和工作思路，解放思想，干事创业，开拓进取，扎实工作，开创了全市经济和社会事业快速发展的新局面。

——经济综合实力明显增强。全市实现生产总值572.69亿元，比上年增长17%，其中一、二、三产业分别增长5.9%、24.3%和14.2%。人均生产总值达到1224美元。全市境内财政总收入45.4亿元，增加7.15亿元，其中地方财政收入22.43亿元，按可比口径增长23.34%。财政支出40.56亿元，增长21.92%。

——经济运行质量效益明显提高。全市规模以上工业实现销售收入692.75亿元、利税69.84亿元、利润42.99亿元，分别增长49.88%、49.72%、45.54%，综合经济效益指数达到178.77，提高24.04个百分点。

——结构调整迈出新的步伐。全市三次产业比例达到20.3∶54.9∶24.8，第二产业比重提高4.6个百分点。林牧渔业占农业增加值的比重达到22.26%；高新技术产业产值占规模以上工业产值的比重达到16.02%，提高1.62个百分点；民营经济增加值占生产总值的比重达到35.25%，提高7.21个百分点；外贸依存度达到7.58%，提高0.55个百分点。

——固定资产投资有较大增长。全社会固定资产投资完成267.38亿元，增长33.1%。其中工业投入182.83亿元，占全社会固定资产投资的68.38%，增长42.6%。

——城乡面貌发生显著变化。全市城市基础设施投资13亿元，其中聊城城区8亿元。全市交通投入13.71亿元，增加5.21亿元；电力、热力生产供应业投入17.19亿元。新增城市道路面积190万平方米；城市绿化覆盖率为34.94%，城市化水平达到31.9%，分别提高1和1.5个百分点；城区空气质量良好率达到94.5%，提高1.4个百分点。

——各项社会事业全面进步。全年共取得重要科技成果108项，新增省级工程技术研究中心1家。初中升入高中阶段学生比例达到61.6%，提高5.35个百分点；高考本科上线12320人，增加4332人。人口自然增长率为5.11‰。文化、卫生、体育、广播电视等事业都取得新的成绩。

——人民生活水平有新的提高。全年城镇新增就业再就业人员7.2万人，城镇登记失业率控制在4%以内。农民人均纯收入3066元，增长14.5%，为1997年以来最高增幅；城镇居民人均可支配收入7665元，增长12.7%；社会消费品零售总额127.32亿元，增长15.25%；城乡居民本外币储蓄余额320.7亿元，比年初增加33.26亿元。

——社会主义政治文明、精神文明建设全面推进，“平安聊城”建设取得新的成效，全市政治安定，社会稳定，人民群众安居乐业。

回顾一年来的工作，我们突出抓了以下几个方面：

（一）认真贯彻中央宏观调控政策，积极应对新的挑战。把宏观调控作为加快发展的机遇，执行政策认真坚决，谋求发展奋发有为。按照中央“区别对待、有保有压”的要求，抓住主要矛盾和关键环节，抑制经济运行中的不稳定、不健康因素，对各类园区进行清理整顿，认真规范土地市场秩序。积极优化投资结构，加强对农业、工业技术改造、基础设施、社会事业、经济适用房等领域的投资，坚决制止低水平重复建设和盲目投资，及时解决资金、土地供应、煤电油运和原材料紧张等突出问题，确保了经济健康运行。

（二）狠抓招商引资，努力增加投入。把招商引资作为推动经济发展的总抓手，先后组织了赴日韩、港台和南方8个城市招商引资活动，取得丰硕成果。全年招商到位资金103.67亿元，与国内外大集团、大公司的合作取得新的突破。始终把增加有效投入作为关键措施，集中抓了611个投资过千万元的项目建设，完成投资190.94亿元，其中过亿元的项目64个，完成投资80.8亿元。聊城华润退城进园、中通客车底盘、冠洲集团60万吨冷轧板一期工程、临清奥博特铜管二期工程、阳谷电缆集团超高压电缆、时风轻卡汽车、冠星纺织10万纱锭等一大批项目相继建成投产。信发集团40万吨氧化铝粉、希杰集团生物工程、鲁西化工工业园等重点项目进展顺利。济聊路与国道309连接线、开发区长江路、连接“391”工程的机场路已竣工通车，薛馆路黄河大桥至聊城段、国道105德聊界至东阿、蒙馆路莘县至冠县段等基本完成。水利、通信、电网改造等基础设施建设都取得了新的成绩。

（三）全力建设百亿产业，推动工业经济快速发展。积极实施工业带动战略，下大力培植支柱产业，积极促进大企业集团的发展，带动产业集群形成和中小企业发展，工业经济保持强劲的发展势头。百亿产业核心企业实现销售收入、利税、利润占规模以上工业比重分别达到47.49%、51.64%、55.72%，比上年提高了27.77、10.58和9.9个百分点。全市实现利税过千万元的企业102户，比上年增加39户，其中过亿元的14户，比上年增加3户。

（四）把“三农”作为重中之重，积极促进农村发展和农民增收。认真落实胡锦涛总书记来聊视察时重要讲话和中央1号文件精神，实行严格的耕地保护制度，强化农田水利基础设施建设，落实“三补一减政策”，加快农村经济结构调整，农村经济发展呈现多年未有的好形势。给农民粮食直补资金达7842万元，减免农业税2.3亿元。新增农业贷款12.91亿元。粮食总产327.23万吨，比上年增长11.93%。棉花、蔬菜、瓜果、畜牧和水产业全面增长。农业产业化水平进一步提高，农产品出口增长42％。积极实施科技特派员工程，450多名科技特派员在农村经济第一线发挥了重要作用。

（五）着力培植“三个亮点”，不断增强发展活力。把外经外贸作为第一亮点来抓，取得明显成效。全市新批外商投资项目63个，比上年增长28.6%；实际利用外资1.68亿美元，增长51.4%。狠抓出口产品结构调整，大力组织机电、纺织产品出口。全市实现进出口总值5.24亿美元，其中出口3.64亿美元，分别增长35.8%和56.5%。对外交往更加广泛，与韩国光明市建立了友好城市关系。大力发展高新技术产业，在北京等七城市开展了引进高校院所科技成果洽谈会和回访活动，成功举办了’2004中国聊城新材料专家高峰论坛。全市高新技术企业达到43家，比上年增加15家。有4个项目进入国家“863”计划、1个项目进入国家攻关引导计划。高新技术创业服务中心建成投入使用。大力发展民营经济，初步培植了轴承、铝制品、密度板、农机配件、农副产品加工等五大特色产业，民营经济实现增加值、利税分别增长62.7%和51.4%。

（六）狠抓城市建设和服务业发展，增强区域发展的辐射拉动力。坚持把城市化和工业化同步推进，把服务业作为经济工作的重点来抓。加强城市基础设施、亮点工程建设，加大环境综合整治力度，城市面貌发生明显变化。创建国家卫生城取得重要阶段性成果，城市人居环境明显改善。水城广场全面竣工，运河博物馆、运河开发三期、人民广场等工程进展顺利。东昌路拓宽改造和聊堂路、聊阳路、光岳路等城市出入口景观改造工程基本完成。城区道路、供排水、供气、供热、供电等基础设施进一步完善。谭庄水库建成运行，从根本上保证了东昌湖、古运河水源补给的持续稳定，对改善江北水城的生态起到了关键作用。八县（市、区）和开发区全部用上管输天然气，成为山东省第一个实现县县用上管输天然气的地级市。县市城区城镇规划建设管理水平全面提升，小城镇建设取得新进展。着力打造以香江大市场、新东方广场、人民广场、金鼎商厦、新时代广场为中心的城市商业区。着力培植旅游支柱产业，运用市场手段成功举办了文化旅游节和水城之秋观光周活动，接待国内外游客385.18万人次，旅游总收入18.48亿元，比上年增长48.1%。高度重视金融、保险业的发展，驻聊金融机构达到8家，保险公司13家。全年金融机构存贷款余额分别为459.04亿元和372.44亿元，比年初增加48.14亿元和45.54亿元；保险费收入9.6亿元，增长29.84%。

（七）关心群众生活，认真负责地为群众办实事。认真落实就业再就业政策，增加就业资金投入，全年新开发就业岗位6.86万个，输出劳务新增15.8万人。加强社会保障体系建设，积极推进扩面征缴，全市用于社会保障的财政支出达4.25亿元，全年共发放“两个确保”资金3.96亿元，发放“低保”资金2704万元，确保了失业人员和困难群众的基本生活。多层次医疗保障体系初步建立，参保人员新增5.86万人，为1.77万人支付医疗保险金8100万元。认真解决拖欠农民工工资问题，清理历年拖欠工资8841万元。抓好农村中心中学、中心小学建设，投资8459万元新建改造校舍13万平方米；积极推进新型农村合作医疗试点，参保农民达49.82万人。加快实施农村道路“村村通”工程，投资5.8亿元新修改造乡村道路4052公里，使87.5%的村庄实现了通油路。完成农村饮水工程54处，又有13万人解决了饮水困难。高度关心农村困难群众的生活，对局部受灾地区及时拨付救灾资金。对农村特困户积极进行救助，发放资金247.1万元、粮食464万斤，救济特困户和低保对象9.59万人。在市级财政比较困难的情况下，安排2.46亿元资金，支持县乡发展。其中，用于转移支付2693万元，社会保障1.42亿元，救灾500万元，农村教育、卫生、水利、交通等基础设施建设4075万元。实施了强弱乡镇改革发展试点。加大扶贫攻坚力度，使6.2万人实现脱贫。积极整顿规范市场秩序，确保物价稳定和食品、药品安全。

（八）坚持统筹协调发展，推进各项社会事业全面进步。基本确立了以县为主的义务教育管理体制，全面实施义务教育“一费制”，积极推行民建公租和后勤社会化改革，职业教育、民办教育和成人教育发展加快。加强公共卫生“两个体系”建设，第一批乡镇中心卫生院改造基本完成。加强文化设施建设，水城明珠剧场、聊城杂技团暨中国少儿杂技基地、聊城京剧团、聊城豫剧团等工程全面竣工使用。群众性体育健身活动更加普及，竞技体育水平不断提高。人口和计划生育工作取得新成绩，东阿、茌平两县被评为全省计划生育优质服务先进县。全面启动生态市建设和创建国家环保模范城工作，在全省城市环境综合整治定量考核中位居第三名。加强国防动员、民兵预备役和人民防空工作，深入开展“双拥”共建活动，积极支持“391”国防工程建设。新闻出版、广播电视、社会科学、民族、宗教、外事、侨务、档案、史志、对台、人防、气象、地震、残疾人、慈善、老龄和妇女儿童等项工作都取得新的进展。

（九）加强政府自身建设，努力改善发展环境。按照“为民、务实、清廉”的要求，大力推进阳光政府、责任政府、服务政府、法治政府建设。认真学习实施《行政许可法》，加强对机关工作人员的培训，对行政许可事项、许可主体及许可收费进行了全面清理，不断规范行政服务中心的运作，优化行政审批环境。积极推进政府科学民主决策，制定出台了《聊城市人民政府工作规则》。加强电子政务建设，开通了聊城政务公众信息网，扩大了政府公报的发行。进一步加强廉政建设，严肃查处各类违法违纪案件，纠正部门和行业不正之风，注重从源头上预防和治理腐败。全面推进“平安聊城”建设，深入开展“严打”整治斗争，加强社会治安综合治理，各类案件明显下降。认真做好信访工作，妥善处理人民内部矛盾，信访总量明显下降。广泛开展“安全生产落实年”活动，加强安全生产专项整治，避免了重特大事故的发生。全市上下安定和谐，发展环境进一步改善。

各位代表，经过全市上下的共同努力，市十四届人大二次会议确定的目标任务胜利实现，“十·五”计划确定的主要指标提前一年完成。我们在全面建设小康社会的征程上又迈出了坚实步伐，取得了重大成就！

各位代表，过去的一年，在面临许多新矛盾、新挑战的情况下，我们能取得这样的好成绩，创造这样的好局面，确实很不容易。这是全市各级认真实践科学发展观、坚决落实中央和省各项决策部署的结果；是中共聊城市委科学决策、正确领导，市人大有效监督、大力支持，市政协积极参政议政、民主监督，社会各界共同努力的结果；是全市人民同心同德、干事创业、辛勤劳动的结果。在此，我代表市人民政府，向全市工人、农民、知识分子、各级干部，向各民主党派、工商联、无党派人士和社会团体，向驻聊人民解放军、武警部队官兵、公安干警和中央、省驻聊单位，向所有关心和支持聊城发展的国内外朋友，向在我市创业的国内外投资者、合作者，表示衷心的感谢和崇高的敬意！

各位代表，在回顾过去一年工作的时候，我们也清醒地看到，当前我市经济和社会发展中还存在不少矛盾和问题，主要是经济的结构性矛盾仍然比较突出，产业层次低，科技创新能力不强，特别是服务业发展很不适应经济社会发展的需要；投资总量相对较小，投资结构不尽合理，主要是大项目、高新技术项目较少；农业的基础还不稳固，促进农民增收的新型机制尚未建立；各项改革推进不快，体制机制性障碍比较严重；对外开放水平不高，经济的外向度较低，利用外资和外贸出口仍处在全省靠后位置；社会事业发展任务很重，与群众物质文化生活的需求还很不适应；社会保障能力不强，就业再就业任务很重，社会生活中还有许多不公平现象，困难群体的许多实际问题还未得到解决；区域经济发展不平衡，部分县乡财政比较困难；政府工作还有不少缺点和不足，有些政府工作人员依法行政的观念不强，工作效率不高、作风不扎实的问题比较突出，一些行业和部门还存在不正之风，发展环境亟需进一步改善，腐败现象在个别地方和单位还时有发生。我们一定要正视这些问题，采取有力措施，认真加以解决。

二、2005年政府工作的总体要求

2005年是贯彻落实科学发展观、巩固宏观调控成果、保持经济社会良好发展态势的关键一年，是全面完成“十·五”计划、为实施“十一·五”规划奠定基础的重要一年。综合分析今年的形势，我市经济社会面临着良好的发展机遇和条件。世界经济将持续增长，国家将继续加强和改善宏观调控，经济社会发展的大环境将进一步改善。经过近几年的不断积累，我市已经具备了良好的发展基础，特别是随着一大批新的增长点的形成，决定了我市经济社会发展在一定时期内会保持较高的增长速度。因此，我们必须抓住机遇，乘势而上，坚定信心，奋力开拓，努力把今年的各项工作做得更加扎实、更加富有成效。

根据省委、省政府的部署和市委的要求，今年政府工作的总体要求是：坚持以邓小平理论和“三个代表”重要思想为指导，认真贯彻党的十六大、十六届三中、四中全会和中央、全省经济工作会议精神，全面落实科学发展观，牢牢把握发展这个第一要务，紧紧抓住重要战略机遇期，坚定不移地实施省、市委工作会议确定的发展目标和工作思路，切实把工作重点放到调整经济结构、深化体制改革和转变经济增长方式上来，突出抓好招商引资、增加投入、工业经济、“三农”工作、“三个亮点”、繁荣三产、城市建设、改善群众生活等主要工作，大力发展各项社会事业，大力加强精神文明和民主法制建设，促进经济社会全面协调可持续发展，全面完成“十·五”计划确定的各项目标任务，为实施“十一·五”规划奠定坚实的基础。

今年全市经济和社会发展的主要预期目标是：生产总值增长15%；地方财政收入增长18%；实现城镇新增就业6万人以上，城镇登记失业率控制在4%以内；社会消费品零售总额增长15%；居民消费价格总水平涨幅控制在4%左右；人口自然增长率控制在7.5‰以内；城镇居民人均可支配收入和农民人均纯收入分别增长8%和6%。实际工作中要加倍努力，力争发展得更快更好。

做好今年的工作，我们必须坚持以科学发展观统领全局，把科学发展观贯彻到经济社会发展的各个方面，切实把握好以下八点：一是准确把握形势。要善于观察形势，认清世界经济走势和产业转移趋势，吃透国家宏观经济政策，增强机遇意识，积极抢抓发展机遇。提高对宏观形势的预见力，善于做好结合文章，创造性地开展工作。二是首抓结构调整。坚持以结构调整为主线，下大气力抓好产业结构、投资结构、财税结构、金融结构、消费结构、所有制结构等一系列重大结构的调整，提高区域经济的综合竞争力。三是努力扩大投资。把扩大投资作为长期的根本性措施，继续下大力抓好招商引资，积极承接国际、国内产业转移，靠有效的投入拉动和促进经济快速发展。四是积极推进改革。强化改革创新意识，认真解决制约经济社会发展的体制机制性障碍，依靠体制和机制创新创造发展新优势。五是全面扩大开放。进一步解放思想，更新观念，全方位推进对外开放，以开放促调整、促改革、促发展，推动全市经济发展上水平。六是突出质量效益。把提高质量效益作为经济工作的中心环节，切实转变经济增长方式，努力提高经济增长的质量和效益。七是高度重视财税。围绕增加财税抓经济、抓投入、抓发展，确保随着经济的增长，财政和税收有较大的增长，增强各级政府的财力，增强解决实际困难的能力。八是统筹协调发展。在加快经济发展的同时，更加重视社会事业的发展，更加重视区域协调发展，更加重视人与自然协调发展，不断提高人民群众的物质文化生活水平，努力构建社会主义和谐社会。

三、2005年经济社会发展的主要任务和工作重点

全面完成今年经济社会发展目标任务，要着力抓好以下十项重点工作。

（一）加快推进经济结构调整，切实转变增长方式，努力提高区域经济整体素质和综合竞争力。调整优化经济结构是一项长期性、战略性、全局性的重要工作，是转变增长方式、参与国际竞争、增强综合实力的迫切需要。要紧紧抓住结构调整不放，全面推动结构优化升级。

加快推进工业结构调整。坚定不移的实施工业带动战略，以培植“一批支柱产业、一批大企业集团、一批知名品牌”为目标，加大工业投入，推进企业重组，拉长产业链条，形成产业集群，再造我市工业经济的新优势。要加快推进百亿产业建设，重点培植电力、有色金属、农业装备、汽车、造纸、新材料等产业，突出抓好核心企业发展，积极实施“5513”工程，争取20户企业销售收入过10亿元，其中4户企业过30亿元，2户企业过百亿元，力争利税过亿元的企业达到18户。要把现有企业做强做大，特别是“5513”工程企业要在领办工业园区、与国内外大企业的合资合作上实现大的突破。筛选一批潜力大、带动作用强的产品作为全市重点培植品牌，力争在三、五年内培植时风、阿胶等8个左右的“中国名牌”。完善鼓励创新的政策体系，提高企业自主创新能力。加大企业改制重组力度，迅速膨胀企业规模。切实抓好中小企业的发展，建设一批“精、专、强”的中小企业群体。借助西气东输机遇，更好地利用中原油田天然气资源。切实抓好工业经济的运行，及时解决资金、土地供应和煤电油运等制约因素，保持工业经济的健康发展。

大力繁荣发展服务业。以旅游业、商贸流通、现代物流业为重点，进一步提升服务业层次和水平。坚持“政府主导、社会参与、市场运作”，发展大旅游，开发大市场，建设大产业，尽快把聊城培育成为全国旅游热点城市，建设成为济南的西花园、京津的度假村。突出“江北水城”和历史文化名城特色，整合旅游资源，挖掘文化资源，精心开发大景区、大景点，力争把江北水城风景区打造成为“4A”级旅游风景区。今年重点抓好两河一湖的贯通，开发夜游水城项目，改建东昌湖沿岸，建设旅游信息服务中心。积极筹办“京津冀鲁豫晋十几城市旅游业发展论坛”，加快构建旅游经济合作区。大力发展假日经济，进一步提升文化旅游节等重大节庆活动的档次和水平。加强配套设施建设，吸引游客延长驻留时间。以建设区域性商贸中心城市为目标，加快发展现代物流业。按照“科学规划、合理布局，调整结构、整合资源，加强监管、有序竞争，改善管理、提高效益”的原则，抓好商贸流通业调整，努力拓宽发展领域和空间，增强商贸流通企业的经营活力和市场竞争力。集中打造城市中心商业区和次中心商业区，在聊城城区着重建设好以新东方广场、金鼎商厦、五星百货、人民广场地下商城为核心的市中心商业区，以香江光彩大市场为核心的城区北部物流园区，以汽车产业园为核心的城区东部物流带，以新时代广场、铁塔商场为核心的城区南部商业区。积极吸引国内外知名物流企业入驻聊城，发展连锁经营，打造市场品牌，构筑交通运输、仓储配送、信息网络等功能配套的现代物流体系。加强社区商业服务设施建设，方便人民群众的生活需求。加快并规范发展交通、通信、房地产和广告、科技、会计、律师、金融、保险等服务业，全面提升第三产业发展水平。

努力推进财税金融结构调整。大力发展对地方财政贡献率高的二、三产业，发展高附加值、高创税的产业，不断提高财政收入占生产总值的比重，提高税收占财政收入的比重。坚持依法征税，完善征管体制，强化税源控管，建立相关部门协税工作机制。大力培植县乡财源，逐步解决县乡财政困难。调整财政支出结构，建立公共财政，把更多的财政资金用到保工资、保稳定、保法定支出、发展公益事业上来。全面整合政府各项财力，集中力量办大事。加快预算编制改革，完善国库集中支付、政府采购、收支两条线制度，对政府组成部门全部实行集中支付。金融结构调整，要进一步强化金融对地方经济社会发展的支持力度，扩大直接融资比重，降低间接融资比重，增加长期融资比重，降低短期融资比重。大力发展信用证、商业汇票、保函、保理等中间业务。优化信用环境，巩固和加强相互依存、共同发展的良好银企关系，引导金融机构拓展金融产品，改进金融服务，增加信贷投放。争取外资银行、股份制银行业务尽快进入聊城。

积极扩大消费需求。健全与经济发展水平相适应的收入增长机制，努力增加城乡居民收入，进一步提高购买力。尽快取消抑制消费的政策，规范和减少行政事业性收费。采取措施引导消费本地产品，积极培育住房、汽车等高端消费，推动消费结构升级。大力发展旅游、文化、娱乐、健身、购物等热点消费，吸引济南等周边地区消费人群进入聊城。大力繁荣城乡贸易，拓宽消费品进入农村市场的渠道，扩大农村消费。积极推广信用消费，促进消费方式转变。加强市场秩序的整顿，切实保护消费者的权益。加强物价监督和执法，努力保持价格总水平的基本稳定。

（二）下大力抓好招商引资，努力增加有效投入，切实提高投资的质量和效益。继续把招商引资、增加有效投入作为经济工作的总抓手，以全新的思维进行谋划，以更加有效的手段狠抓落实，确保招商引资和固定资产投资实现新突破。

千方百计抓好招商引资。今年招商引资到位资金要达到120亿元。一是选准招商重点。继续把韩国、日本和香港、台湾作为招商重点区域，重视欧美、东南亚地区的招商。巩固与拓展国内8个城市的招商成果，实现长三角、珠三角地区招商引资的新突破。二是创新招商方式。继续打造招商平台，适度安排大型招商活动，集中精力抓好小团组招商、专业队伍招商、网上招商和委托招商。组建高层次、高水平、高素质的招商队伍，分区划片搞好招商。三是选好招商载体。整合招商资源，围绕优势产业和大企业集团进行招商，围绕产业集群和产业链进行招商，围绕农业、旅游资源开发利用进行招商。充分发挥聊城经济开发区招商主载体作用，举全市之力办好开发区。四是注重招商质量。外商来聊发展，必须在聊城设立独立法人，以对地方发展的贡献确定优惠政策。严禁引进高污染、高耗水、高耗能项目。

努力增加有效投入。要围绕结构优化和发展高新技术产业抓投资，围绕增加财政收入、增加社会就业、增加农民收入抓投资，围绕城乡协调和可持续发展抓投资。集中力量扶持一批科技含量高、带动作用大的高新技术产业和制造业项目，扶持一批重大基础设施和社会事业项目，扶持一批促进服务业和旅游业发展的项目。重点抓好投资10亿元以上的聊城鲁能热电有限公司2×300MW热电机组项目、国电中华电力聊城发电厂二期工程、华信铝电集团40万吨氧化铝、泉林纸业林浆一体化项目和投资亿元以上的韩国希杰集团生物科技、景阳冈冷轧钢板有限公司100万吨冷轧钢板、侨润集团10万立方米德国莱明板、凤祥集团乳制品、冠州集团60万吨冷轧板和蓝山集团生猪养殖宰杀及系列熟肉制品开发等一批工业项目。全面完成青银高速公路聊城段的建设，开工建设聊城至高唐等5条干线公路，积极争取德商高速、薛馆高速尽快开工建设。加强和完善重点项目管理、调度制度，力争尽快建成投产，发挥效益。做好项目的筛选、储备工作，争取一批重点项目列入国家和省“十一·五”规划。

（三）以增加农民收入为中心，切实抓好“三农”工作，促进农业和农村经济快速健康发展。坚持城乡统筹发展，以工促农，以城带乡，积极推进“四个配套联动”，紧紧抓住结构调整、产业化经营、农产品流通等关键环节，促进农业增产和农民增收，保持农业和农村经济发展的好势头。一是提高粮食综合生产能力。实行最严格的耕地保护制度，加强基本农田保护。启动实施“沃土工程”，加强农田水利建设。抓好优质粮食基地建设，加快农业科技创新和技术推广，确保粮食播种面积和粮食总产的基本稳定。二是进一步调整优化农业结构。坚持以市场为导向，以提质增效、规模经营为重点，引导农民发展畜牧、水产、蔬菜、水果、林木、花卉等高效农业，发展适销对路的特色种养业。加快建设优质农产品产业带，优化农业区域布局。完善农业标准体系和农产品质量监测、认证体系及动物防疫体系，启动优势农产品竞争力提升计划，不断提高农产品国际竞争力。大力推进农业产业化经营。瞄准市场需求，加快推进畜牧、林业、粮油、果品、蔬菜的产业化。围绕产业化，大力实施“五个一批”工程，即：着力培植一批农副产品加工龙头企业，培植一批特色产业基地，培养一批有技术、会管理的农民种养大户，培养一批熟悉市场、善于经营的农民运销组织，选派一批敢闯敢干、带领农民致富的科技特派员。三是下大力抓好农产品流通。继续发育好本地市场，在聊城城郊建设一处大型农产品批发中心。努力开拓国内外高价位市场，将我市的农副产品打入京津济沪宁等大城市批发市场和超市，使其成为著名的品牌。大力发展农产品运销队伍，建立新型农民合作组织或行业协会，为广大农民搞好服务。与周边省市协调，开辟绿色通道，确保货畅其流。四是加快农村劳动力转移。加快小城镇建设和农村二三产业发展，引导更多的农村劳动力实现就地转移。整合现有培训资源，重点扶持一批职业培训机构，形成市场——订单——培训——输出的新型劳务协作方式，力争今年劳务输出新增10万人以上。维护农民工的合法权益，取消对进城务工人员的歧视性政策，切实解决好拖欠和克扣农民工工资等问题。五是全面落实扶持农业发展的各项政策。深化农村税费改革，今年农业税税率再降低2个百分点。进一步落实好对种粮农民直补、良种补贴和购置大型农机具补贴政策。市县财政预算安排支农支出都要有较大增长。切实加强监管，严格防止农民负担反弹。六是继续推进农村各项改革。稳定和完善土地承包制度，推进农村基础设施产权多元化，积极探索农业规模化、集约化经营的新途径和新模式。大力推进乡镇配套改革。放开放活粮食市场，搞好粮食流通体制改革。

（四）着力抓好“三个亮点”，营造发展新优势，进一步增强经济发展活力。外经外贸、高新技术、民营经济是我市经济发展的潜力所在、希望所在、活力所在，要做大做强“三个亮点”，力争实现更大的突破。

全方位提高对外开放水平。努力扩大利用外资规模，提高利用外资质量，力争利用外资增长40%以上。要引导外资更多地投向高新技术、先进制造业、现代农业、现代服务业、环保产业，鼓励参与国有企业改组改造。引导企业搞好引进技术的消化吸收和创新提高，进入跨国公司的全球生产、销售网络。以园区和大企业为主要载体，努力突破与世界500强合资合作，重点抓几个利用外资过5千万美元的大项目。积极推动外贸出口，力争出口额增长40%以上。大力调整出口产品结构，巩固传统产品出口，着力扩大机电产品，特别是扩大汽车、农用车、发动机的出口。今年着力抓好几个出口过亿美元的大县和过亿美元的大企业。深入实施科技兴贸和以质取胜战略，鼓励企业获得国际通行的产品质量、环境管理和安全标准等体系认证。大力发展现代加工贸易，积极吸引跨国公司把技术含量高、增值大的加工环节转移到我市。加快外贸主体多元化，赋予更多民营企业和中小企业进出口经营权。认真落实出口退税政策，保护和调动出口企业的积极性。完善外经贸预警机制，提高处理国际贸易争端的能力。加快实施“走出去”战略，鼓励有实力的企业到境外办厂、开展工程承包和劳务合作。切实保护在外务工人员安全。提高“大通关”服务水平，增强环境吸引力和竞争力。

加快发展高新技术产业。坚持以信息化带动工业化，突出发展电子信息、生物技术和制药、新材料三大高新技术产业，确保高新技术产业产值占规模以上工业产值的比重提高2个百分点。加快运用高新技术和先进适用技术嫁接改造传统产业。积极促进大企业与高校院所联合建立实验室和研发中心，提高自主知识产权创新能力。组织好聊城科技合作论坛和引进高校、科研院所成果5周年展览活动。充分发挥市创业服务中心的平台作用，加快建立市级高新技术产业园区。建立风险投资机制，形成以政府投资为引导、企业投资为主体、金融和风险投资为支撑的多元化高新技术投融资体系。

实现民营经济发展新突破。创造平等竞争、一视同仁的法制环境、政策环境和市场环境，推动民营经济扩大规模、提高水平、提升竞争力。按照政府引导、市场运作的原则，建立民营经济融资担保、信用评价、人才培训、技术信息、法律服务和行业协会6个体系。突出抓好100户重点民营企业和14个民营经济园区建设。鼓励民营企业进入更多的领域和行业，参与国有和集体企业的改制与重组。引导有条件的民营企业建立现代企业制度，提高经营管理水平。

（五）大力实施“精细”工程，加快城市建设步伐，营造更好的创业和人居环境。加快推进城市建设由低层次、低水平、粗放式向高品位、高质量、精细化的转变，坚持高起点规划、高标准建设、高效能管理，着力在精细建设、精细管理上下功夫，在创造精品上下功夫。一是高水平搞好城乡规划。把聊城城区的详细规划做精、做深、做出水平，尽快完成临清市和六个县城的规划修编。搞好镇、村规划，每个县（市、区）都要抓一批示范村的规划，以此带动村庄规划建设上水平。二是加快城市基础设施和重点工程建设。完成运河三期工程，开工建设运河四期工程，实现运河与徒骇河的贯通，完善江北水城主体框架。抓好人民广场、南关岛、月亮岛等工程建设。进一步抓好城市供水、供热、供气、公交、垃圾和污水处理等设施建设，抓好兴华路、站前街等道路的改造，完成日处理能力400吨的生活垃圾处理厂建设，加快第二污水处理厂的建设。三是搞好城市环境综合整治。继续抓好国家卫生城的创建，全面改善城市环境，彻底解决扬尘过多、违章建设、乱停车辆、占道经营、乱摆摊点、三轮车过滥等问题。突出抓好背街小巷和城中村的改造和整治。大抓绿化、美化，努力增加绿量，提升绿化档次，营造绿色家园。改进城市管理方式，理顺管理体制，推动管理重心下移，建立长效机制，保持良好的市容市貌。四是切实搞好城市经营。重点抓好土地经营、城市资源经营，依法规范土地出让，实现土地收益6.5亿元，其中城区2.7亿元。五是加快城镇建设。抓好临清和六个县城以及中心镇的建设，引导产业和人口向城镇集聚，城市化水平达到34％。

（六）坚持统筹兼顾，注重协调发展，全面发展各项社会事业。坚持统筹经济社会发展，切实把科技教育摆在优先发展的战略位置，把人才资源作为第一资源，全面发展各项社会事业。

大力实施科教兴聊和人才强市战略。一是切实加强科技工作。尽快制定并完成中长期科技发展规划。切实增加对科技的投入。进一步深化科技体制改革，充分调动科技人员的积极性。加强企业技术研发中心建设，大力发展各类科技中介服务组织。继续加强与国家“863”计划的对接，争取更多的项目列入国家科技发展计划。强化信息基础设施建设。加强知识产权保护。高度重视社会科学研究，搞好成果的实际运用。二是把教育放在优先发展的战略地位。进一步强化学前教育和基础教育，巩固提高“普九”水平，加快发展高中阶段教育，努力提高教育质量。继续推进农村中心中学、中心小学建设，进一步改善办学条件。积极倡导民建公租和后勤社会化改革，鼓励民间资金投资教育事业。搞好职业教育资源配置和调整。大力支持高等教育发展，为聊城大学、聊城职业技术学院创造良好的办学环境。三是大力实施人才强市战略。抓住培养、吸引、使用三个环节，加强人才资源能力建设。加强企业家队伍建设，面向全国招聘优秀企业经营者、管理者和技术人才，推进企业体制、机制、管理和技术创新。加强高级技工队伍建设。深化人才制度改革，建立鼓励优秀人才脱颖而出、人尽其才的有效机制和良好环境。

加快发展卫生、文化、体育、计划生育等各项社会事业。突出抓好公共卫生建设，全面完成市县两级疾病预防控制体系和医疗救治体系建设，切实抓好传染病防治工作；加强农村卫生基础设施建设，完成第二批乡镇中心卫生院改扩建工程；抓好农村新型合作医疗试点工作。加强文化大市建设，发掘保护和利用传统文化资源，推进文化事业产业化；完成中国运河文化博物馆的建设；大力发展城市社区文化，办好乡镇文化中心和农村文化大院；加快农村有线电视入户步伐。发展竞技体育，积极备战“十运会”，举办第一届“市运会”，积极开展全民健身和群众性体育活动；抓紧搞好聊城市体育中心的规划和选址，适时开工建设。坚持人口与计划生育目标管理责任制，努力平抑第四次人口出生高峰，稳定低生育水平，优化出生婴儿性别结构，提高人口素质。切实搞好国防教育、国防动员、人民防空和民兵预备役建设，做好军队转业干部、退役士兵安置工作，积极支持驻聊部队建设，更加扎实地开展双拥共建活动。大力做好广播电视、新闻出版、民族、宗教、外事、侨务、对台、档案、史志、气象、防震、救灾、妇女儿童、老龄以及慈善事业、残疾人等项工作。

促进区域经济协调发展。认真探索研究融入济南都市圈的战略规划，主动接受济南辐射，创造区域发展合作的新优势。高唐、临清、茌平要自我加压、奋力赶超，及早进入全省、全国强县市行列；东昌府、阳谷要加快发展，位次前移，实现大的跨越；继续加大对莘县、冠县和东阿的支持力度，三个县要充分利用省里的帮扶政策，启动内力，努力取得突破性发展。加快推进15强和16弱乡镇改革发展，选准突破口，实现强者更强，弱者赶上。切实抓好马西地区的帮扶工作，使其尽快改变落后面貌。

（七）坚持以人为本，关心群众利益，努力提高群众生活水平。牢固树立以人为本的思想，倾注真情为人民群众办实事、解难事、做好事，使广大人民群众更多地享受经济社会发展成果。一是努力扩大就业。把劳动密集型制造业、民营经济、服务业、中小企业和社区作为扩大就业的重要领域。全面落实各项就业扶持政策，建立促进就业增长的长效机制。推进“公益性岗位开发计划”和“再就业伙伴计划”。大力发展职业培训，提高下岗失业人员和进城务工农民的创业、就业能力。二是完善社会保障体系。继续巩固“两个确保”，规范和完善城市“低保”工作。扩大社会保险覆盖面，提高个体私营等非公有制企业的社会保险参保率。认真抓好低保标准提高工作的落实，确保低保资金按月足额发放。严格执行最低工资标准，保障职工的合法权益。健全完善以基本医疗保险为主体的多层次医疗保险体系。稳妥推进企业离退休人员社会化管理服务，有条件的县（市、区）基本实现街道社区属地管理。三是做好济困救助工作。更加重视和关心社会弱势群体，建立健全城乡低收入困难群众社会救助体系，建立社会医疗救助制度。进一步完善农村特困户救助制度，逐步实现向农村低保制度的过渡。积极发展慈善事业，做好受灾群众生活救济工作。四是继续实施“安康居住”工程。以解决中低收入和弱势群体的住房困难为重点，建立完善以普通商品房、经济适用房和廉租房为主体的住房供应体系，加大中低价位、中小户型住房建设。五是切实改善群众的生产生活条件。今年再投资4.7亿元，改造农村公路3600公里，确保年底基本实现村村通柏油路。投资2400万元，搞好农村人畜饮水解困工程，再解决113个村、9.3万人的饮水困难。启动村村通自来水工程，力争用三年的时间，使全市65%以上的村庄用上安全卫生的自来水。投资2.2亿元，改造农村电网设施；投资3.15亿元，新建改造农村的水利基础设施。财政投资4000万元，搞好农村中心中小学建设。安排农村中小学助学金和免费发放教科书专项资金，建立学籍管理制度，把辍学率严格控制在2%以内。

（八）着眼于体制机制创新，加快推进各项改革，为经济社会发展提供强大动力。把改革摆到突出位置，既要推进关系全局的重大改革，又要推进局部领域的改革创新，使改革真正成为推动各项工作的动力。一是深化国有企业和国有资产管理体制改革。以建立现代产权制度和内部法人治理结构为重点和方向，加快国有企业股份制改革和战略性重组。规范企业改制，试行职业经理人制度。基本完成市直国有工业、建安、交通运输、商贸企业改制任务。完善国有资产监督管理体系，落实国有资产经营责任，实行严格的经营业绩考核制度，确保国有资产保值增值。二是稳妥推进投资体制改革。逐步建立起市场引导、企业自主决策、银行独立审贷、融资方式多样、中介服务规范、宏观调控有效的新型投资体制。出台投资体制改革实施意见，完善建设项目登记备案办法和核准办法。出台政府投资项目管理办法，引入市场机制，加强运营管理，完善政府投资后评估制度，建立严格的投资决策责任追究制度。鼓励社会资本投资，允许进入未禁止的基础设施、公用事业等领域。三是加快现代市场体系建设。积极发展土地、技术和劳动力市场。建立健全市场准入机制，继续整顿和规范市场经济秩序，严厉打击制售假冒伪劣商品、盗版侵权、偷逃骗税等经济违法犯罪活动。密切关注市场物价动态，防止囤积居奇、物价大起大落。大力推进社会信用体系建设，努力建设“诚信聊城”。四是推进事业单位改革。按照统筹规划、分类改革的原则，逐步建立政事职责分开、法人地位明确、单位自主运营、政府依法管理的新型管理体制和运行机制；建立单位自主用人、人员自主择业的用人制度；建立重实绩、重贡献和生产要素参与分配的分配激励机制。大力推进城建体制改革，继续搞好市政公用事业改革。在抓好上述几项重大改革的同时，全面推进各部门、各单位、各企业自身的改革，特别是在用人机制、分配制度、财务管理、机关管理和后勤社会化改革等方面，创新机制，完善制度，革除弊端，靠改革增活力、增效率、增效益。

（九）加快生态市建设，大力发展循环经济，努力建设资源节约型社会。把建设生态市和发展循环经济作为政府工作的重要任务，建立节约型生产生活方式，实现人与自然和谐发展。

全面落实生态市《规划纲要》。启动碧水行动计划，加强重点流域、区域的污染综合治理，大力发展生态农业，抓好农业面源污染防治。严格执行环境影响评价和“三同时”制度，从源头上控制新的环境污染和生态破坏。全面实施排污许可证制度，建立严格规范的排污总量控制体系。以滩区、沿路、沿河和环城、围村为重点，大力开展植树造林。加强土地管理，盘活存量建设用地，搞好土地复垦开发，提高土地集约利用水平。

大力发展循环经济。系统推进循环经济试点，抓好示范企业、示范园区、示范城市试点。按照减量化、再循环、再使用原则，突出抓好节能、节水、节地、节约原材料工作。推行清洁生产，重点抓好泉林、凤祥、东胶、蓝山等15户重点企业。加强对工业“三废”的回收利用，推广沼气工程和秸杆综合利用技术，提高资源循环利用水平。大力推广资源节约新技术、新产品、新工艺和新材料，努力降低资源消耗和企业成本，争取今年万元产值综合能耗降低3%，重点产品单位能耗指标稳定降低率达到80%。

（十）加强民主政治建设，全力打造“平安聊城”，切实维护社会稳定。各级政府要自觉接受人大及其常委会的监督，认真落实人大及其常委会决议决定。积极支持人民政协履行职能，主动听取各民主党派、工商联、无党派人士及人民团体的意见和建议。认真做好人大代表议案、政协委员提案的办理工作。积极推进政务公开、厂务公开和村务公开。加强农村基层政权建设和城市社区建设。坚持依法治市，深入开展普法教育，提高全民法律意识。全面加强社会主义精神文明建设，大力开展爱国主义教育，扎实推进文明城市、文明村镇、文明行业创建活动，不断提高人民群众的文化素质和社会文明程度。深入推进“平安聊城”建设，坚持工作重心下移，全面落实社会治安综合治理责任制，严厉打击各类刑事犯罪。完善化解人民内部矛盾的工作机制，及时排查调处社会矛盾纠纷，认真做好人民群众来信来访工作，积极预防和妥善处置群体性事件。进一步加强安全生产工作，建立安全生产的长效机制，严防各类重大恶性事故的发生。

各位代表！编制国民经济和社会发展第十一个五年规划，是今年的一项重要工作。各级政府要高度重视，精心组织安排，深入调研论证，广泛征求意见，集中民智民意，力求编制出一个具有较高预见性、科学性、指导性的好规划。

四、加强政府自身建设

完成今年的各项任务，各级政府必须按照“科学执政、民主执政、依法执政”的要求，切实加强自身建设，加快建设责任政府、阳光政府、服务政府和法治政府，全面提高行政能力和工作水平。

（一）科学民主决策，坚持依法行政。建立健全科学民主决策机制，不断提高政府决策科学化、民主化水平。按照“充分论证、公开透明、责任追究、执行反馈”四个环节推进政府科学民主决策。对重大问题及专业性较强的决策事项，要认真组织专家论证和决策评估。对涉及面广、事关人民群众切身利益的决策事项，要实行公示、听证制度，广泛听取群众意见，主动接受群众监督。坚持“谁决策、谁负责”的原则，对超越权限或违反法定程序造成重大损失的决策行为，要严肃追究决策者的责任。各级政府要全面贯彻《行政许可法》和《全面推进依法行政实施纲要》，严格按照法定权限和程序行使权力、履行职责，切实保障人民群众的合法权益。要善于依法管理经济社会事务，做到有权必有责、用权受监督、侵权须赔偿、违法要追究。加强和改进行政执法，做到严格执法、公正执法和文明执法。加快行政执法体制改革，相对集中行政许可权和行政处罚权，推进综合执法。

（二）转变政府职能，提高行政效率。按照“政企分开、政资分开、政事分开”的要求，进一步深化行政管理体制改革，切实把政府职能转到经济调节、市场监管、社会管理和公共服务上来，做到不越位、不错位、不缺位。改进政府管理经济的方式方法，努力提高按市场经济规律管理经济的能力，提高按国际通行规则办事的能力，提高运用经济法律手段调节经济的能力。加快建立健全各种突发事件应急机制，提高应对公共危机的能力。继续深化行政审批制度改革，进一步依法规范行政审批事项。认真抓好市行政服务中心的建设和管理，加快电子政务建设，完善服务功能，提高行政效率。进一步推行政务公开，继续搞好政府网站建设，扩大政府公报赠阅范围，建立政府新闻发言人制度，充分发挥其公开信息、宣传政策、引导舆论、维护稳定的作用。

（三）切实改进作风，狠抓工作落实。要牢固树立以人为本、执政为民的理念，始终保持同人民群众的血肉联系，切实做到权为民所用，情为民所系，利为民所谋，一切工作都要从人民群众的根本利益出发，所有工作的成败得失都要由人民群众的满意度来检验。要大力发扬求真务实的优良作风，说实话，办实事，求实效，坚决反对形式主义、官僚主义，坚决反对弄虚作假、虚报浮夸。严格执行《统计法》、《审计法》，加强对经济工作的审计监督。要大兴调查研究之风，切实精简会议和文件，腾出更多的精力深入基层，抓工作落实。要健全和完善决策目标、执行责任、监督考核“三个体系”，确保工作落到实处。继续办好“96111”热线服务中心，开通市长公开电话受理中心，建立政府与人民群众沟通的快捷通道。

（四）坚持从严治政，狠抓队伍建设。各级政府及其工作人员要认真遵守党的纪律，认真遵守国家法律，坚决贯彻执行中央和省、市委关于廉洁从政的各项规定。按照“标本兼治、综合治理、惩防并举、注重预防”的要求，搞好廉政建设，深入开展反腐败斗争，严肃查处违法违纪行为，严肃惩处腐败分子。各级政府领导要认真落实党风廉政建设责任制，严格履行廉政承诺，以身作则，当好表率，抓好班子，带好队伍。以群众反映强烈的问题为重点，坚决纠正损害群众利益的不正之风，重点解决好征用土地、城镇房屋拆迁、企业重组改制和破产中损害群众利益，以及拖欠农民工工资等问题。要按照市委的统一部署，在政府机关深入开展保持共产党员先进性教育活动，认真解决好思想、纪律、作风以及工作方面存在的突出问题，增强党员队伍的战斗力，提高拒腐防变能力，开创政府工作的新局面。

各位代表！我们聊城正处于实现跨越发展的关键时期。我们肩负的任务光荣而又艰巨。让我们紧密团结在以胡锦涛同志为总书记的党中央周围，在省委、省政府和市委的坚强领导下，解放思想，振奋精神，开拓进取，扎实工作，在改革开放和现代化建设的道路上迈出更大的步伐，取得更大的成就！

让我们团结起来，万众一心，向着新的伟大目标奋勇前进！
